# Industrial Graphene Coating of Low-Voltage Copper Wires for Power Distribution

**DOI:** 10.1021/acsaenm.3c00249

**Published:** 2023-06-01

**Authors:** Neeraj Mishra, Ylea Vlamidis, Leonardo Martini, Arianna Lanza, Zewdu M. Gebeyehu, Alex Jouvray, Marco La Sala, Mauro Gemmi, Vaidotas Mišeikis, Matthew Perry, Kenneth B. K. Teo, Stiven Forti, Camilla Coletti

**Affiliations:** † Center for Nanotechnology Innovation@NEST, Istituto Italiano di Tecnologia, Piazza San Silvestro, 12, 56126 Pisa, Italy; ‡ Graphene Labs, Istituto Italiano di Tecnologia, Via Morego 30, 16163 Genova, Italy; § AIXTRON Ltd., Anderson Road, Swavesey, Cambridge CB24 4FQ, United Kingdom; ∥ Baldassari Cavi, Viale Europa 118/120, 55013 Capannori (Lucca), Italy

**Keywords:** CVD graphene, copper wires, electrical conductivity, oxidation prevention, in-line coating

## Abstract

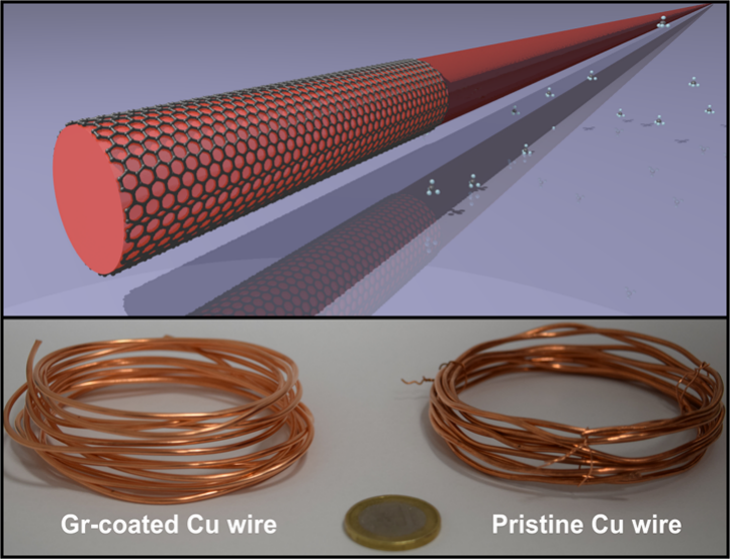

Copper (Cu) is the electrical conductor of choice in many categories of electrical wiring, with household and building installation being the major market of this metal. This work demonstrates the coating of Cu wires—with diameters relevant for low-voltage (LV) applications—with graphene. The chemical vapor deposition (CVD) coating process is rapid, safe, scalable, and industrially compatible. Graphene-coated Cu wires display good oxidation resistance and increased electrical conductivity (up to 1% immediately after coating and up to 3% after 24 months), allowing for wire diameter reduction and thus significant savings in wire production costs. Combined spectroscopic and diffraction analysis indicates that the conductivity increase is due to a change in Cu crystallinity induced by the coating process conditions, while electrical testing of aged wires shows that graphene plays a major role in maintaining improved electrical performances over long periods of time. Finally, graphene coating of Cu wires using an ambient-pressure roll-to-roll (R2R) CVD reactor is demonstrated. This enables the in-line production of graphene-coated metallic wires as required for industrial scale-up.

## 1 Introduction

The applicative realm of copper (Cu) is enormous: it is utilized in indoor and outdoor constructions, tools, machinery, and portable devices, both for civil and industrial uses. In particular, copper wires are commonly employed in industrial wiring and interconnection technology,^1^ owing to their excellent advantages of thermal and electrical conductivity, low cost, and good mechanical properties such as ductility. However, copper wires easily oxidize when exposed to air even at room temperature, waning their electrical performances with time in low-voltage (LV) applications and limiting their use in high-power electronic devices and integrated circuits.^2,3^ Therefore, nowadays, research efforts are devoted to develop effective processes and coatings to prevent oxidation and corrosion of Cu wires, thus preserving the electrical properties of the pristine metal over time.^4,5^ Graphene represents an interesting candidate material for ultrathin coating of Cu wires, thanks to its extraordinarily high electrical and thermal conductivity, flexibility, strength, and chemical inertness.^6−9^ Moreover, graphene’s tight structure is impermeable to gases and liquids,^10,11^ exhibiting remarkable performance as a protective barrier in comparison to other thin-film materials.^12^ A number of papers have discussed the potential of graphene as a multifunctional coating for Cu, hindering oxidation and chemical etching^13−15^ while increasing electrical and thermal conductivity.^16,17^ Concerning Cu wires, it has been demonstrated that multilayer graphene can be synthesized on cylindrical conductors by chemical vapor deposition (CVD),^13^ providing antioxidation protection up to 350 °C and corrosion inhibition in ammonium persulfate solution.^18,19^ Furthermore, graphene-coated (Gr-coated) Cu wires with diameter ≅ 0.1 mm displayed improved surface heat dissipation, electrical conductivity, and thermal stability up to 450 °C.^9,19^ However, to date, no work has demonstrated an industrially compatible approach for this process, *i.e*., performing graphene coating of cables complying with the following requirements: (i) process gases below the lower explosive limit (LEL), (ii) temperatures below 1000 °C, and (iii) ambient pressure. Addressing all of these points would pave the way to the in-line CVD Gr coating of metallic wires by an ambient-pressure roll-to-roll (R2R) CVD system and is the object of our work. Moreover, we address the coating of LV wires, which are expected to generate the highest revenue in the copper wire and cable market in the 2021–2030 timeframe.^20^

Indeed, this work demonstrates that wires with diameters technologically relevant for LV applications (*i.e*., 1.37 and 1.74 mm) can be effectively coated with graphene by a scalable and industrially compatible process: at a temperature below 1000 °C, at ambient pressure, and with rapid growth in a nonexplosive atmosphere. The Gr-coated wires, characterized by electrical measurements and microscopic and spectroscopic techniques, display higher electrical conductivity and improved esthetics than uncoated wires, allowing for a significant saving in the Cu market. Aging of Gr-coated wires is investigated, and in-depth studies to correlate the electrical performances and the microstructural changes after the coating are provided. It is found that the graphene growth process induces an improvement in the crystallinity of Cu wires, which is responsible for the augmented conductivity, and that graphene coating plays a major role in maintaining this improved conductivity over time. Finally, the production of Gr-coated Cu wires with an open-end R2R pilot CVD system is demonstrated. Implementing in-line graphene coating of metallic wires in an industrial setting is required.

## 2 Materials and Methods

### 2.1 Sample Preparation

Industrial LV Cu wires were provided by Baldassari Cavi, a manufacturer of Cu wires operating in the European market.^21^ Rigid Cu wires with diameters of 1.37 and 1.74 mm were used, and these are technologically relevant for LV applications, especially in the Northern Europe. For each wire diameter size, three sets of samples were studied: (i) pristine Cu wires (reference); (ii) Gr-coated Cu wires: annealed at a high temperature (*i.e*., to increase the Cu grain size and reduce the surface roughness and defect density^17^) and then subjected to the graphene growth process; and (iii) only annealed Cu wires (*i.e*., subjected to the same annealing conditions as those used to process wires in point (ii)).

To facilitate the industrial translation of the process, the wires were used as-received for all tests carried out (i.e., no additional pretreatment, such as etching or chemical cleaning was done before processing the samples). Copper wires 2.5 m in length were taken from the bundle and arranged in a coil shape, in such a way that the coil could fit inside a 4 in. BM Pro AIXTRON CVD reactor where wire treatment was performed^22^ (see Figure S1 in the Supporting Information for the reactor setup). The coils were bound with thin, flexible copper wires (diameter 250 μm) to prevent them from unwrapping and thus touching the inner walls of the reactors during heating and cooling steps. Before the growth process, argon (Ar) gas was flushed to purge the reactor from air.

The optimized process was carried out between 900 and 1000 °C under an Ar pressure of 750–800 mbar. Details of the temperature profile and conditions employed in the CVD process are reported in Figure S2. Cu wires subjected only to thermal annealing were placed in the reactor and heated at 980 °C for 10 min in a flow of Ar at 2000 sccm. In the case of Gr-coated samples, after the initial annealing of 10 min, 2 sccm of methane (CH_4_) were flushed for 5 s in hydrogen (H_2_) and Ar (20 and 2000 sccm, respectively) at 980 °C to grow graphene. After either annealing or growth, the samples were cooled down in Ar flow. Processed copper wires were taken out from the reactor once the temperature reached 120 °C. The process conditions were transferred from the batch reactor to the R2R CVD system.

### 2.2 Sample Characterization

Raman spectroscopy was performed to assess the coverage, quality, and number of graphene layers. A Renishaw InVia system, equipped with a 473 nm blue laser, a 100× objective, and an 1800 l/mm grating, was used. Spatially resolved Raman maps were obtained with a 1 μm step while irradiating the samples with 22.3 mJ μm^–2^. The number of graphene layers was determined by analyzing the *I*_2D_/*I*_G_ ratio, where *I*_2D_ and *I*_G_ are the intensities of the 2D and G peaks in the Raman spectrum, respectively.^23^

The surface morphologies of the as-grown and aged wires were investigated by scanning electron microscopy (SEM, ZEISS Merlin), operating in Inlens Signal mode at 5 kV voltage and 120 pA current. Additionally, atomic force microscopy (AFM) was performed with a Bruker Dimension Icon in standard tapping mode. Optical microscopy (Zeiss Axioscope7 equipped with Axiocam 208 color) was employed to record bright-filed micrographs and assess Cu oxidation.

For the X-ray diffraction (XRD) experiments, short wires (2–3 cm long segments) were processed with the same parameters described in Section 2.1, varying only the process time. In particular, analysis was done for samples annealed in Ar for 5, 10, and 30 min and Gr-coated samples annealed/grown for increasing times. To further investigate the effect of hydrogen on the microstructure of copper, analysis was also performed on samples annealed in Ar and then processed in H_2_ flow, employing the same ratio of H_2_/Ar as in the growth process. XRD measurements were carried out on a STOE Stadi P diffractometer equipped with Cu Kα_1_ radiation (λ = 1.5406 Å) and a Ge(111) Johansson monochromator from STOE & Cie. The samples (2–3 cm long wire segments) were mounted on a goniometer head and optically aligned with the diffractometer’s center of rotation and kept under spinning during the measurements. The diffracted intensities as a function of the scattering angle, 2θ, were acquired in the range 84–127° by a MYTHEN2 1 K detector from Dectris. The profiles of the Cu(311), (222) and (400) peaks were analyzed individually, by fitting each of them with a pseudo-Voigt function. The dependence of the peak full- width at half-maximum (FWHM) on the sample treatment conditions was qualitatively evaluated for each of the three peak families and for each of the two wire thicknesses. Due to the bulk nature of the samples and the peculiar experimental geometry, the position and the intensity of the peaks do not directly provide any crystallographic information, but the variation of the normalized FWHM with respect to the pristine material is indicative of the variation in the crystallite strain.^24^

Electrical resistivity measurements on the Cu samples were performed in the DC configuration using a Keithley 2450 source meter. In order to minimize measurement errors, we performed all of the electrical measurements in a four-probe configuration. Such a setting reduces the variation of the contact resistance between the probe and the measurement clamp due to surface oxidation. Each set of samples were tested by putting the source probe at the end of the wires, while the sensing probes were put at exactly 2 m apart through a couple of crocodile clips. The resistance was then measured by applying a constant DC current of 10 mA and measuring the voltage drop between the two sensing tips. Performing measurements in the DC configuration allows us to neglect the skin effect. All measurement were performed at room temperature.” Conductivity was calculated as follows: σ = *LR*^–1^*A*^–1^, where *R* is the resistance of the cable, *A* is its cross-sectional area defined by design, and *L* is the distance between the sensing probes (2 m for our measurements).

Also, additional electrical resistivity measurements were performed at industrial premises (*i.e*., Baldassari Cavi) in a commercial setup that allows measurements at controlled temperature, see Figure S3 of the Supporting Information. A 2.5 m long coiled copper wire was kept inside the water maintained at 20 °C and firmly fixed at both ends so that there was no bending or loops. A couple of wedge holders were used as electrical sensors, while the wire holder at the end of the line provided the source current, with the distance between them that could be precisely tuned. In this way, it was possible to measure the resistivity of pristine and graphene-grafted wires avoiding any variation arising from the copper temperature or contact resistance and reducing the uncertainty on the wire length. In addition, before the resistivity was measured, a small portion of the wire was cut and measured to check the possible changes in diameter before and after the process. For the electrical measurements reported, the average electrical conductivity was obtained from measuring seven samples of each type. The oxidation experiments were conducted within a climate chamber (Espec SH-262 Benchtop Temperature and Humidity Chamber) at temperatures between 40 and 90 °C, humidity levels between 80 and 100%, and times between 20 s and 24 h.

## 3 Results and Discussion

### 3.1 Gr-Coated Cu Wires via Industrially Compatible CVD: Properties

Preliminary studies were performed to identify the process conditions to obtain graphene-coated Cu wires while satisfying the following requirements: (i) process gases below the LEL, (ii) temperatures below 1000 °C, and (iii) ambient pressure. A summary of such preliminary investigations is reported in the Supporting Information (SI) (Figures S4 and S5). Notably, and different from what is reported in the literature until now, the optimized process shown herein has a volume percentage of explosive gases, which complies with LEL requirements. Indeed, H_2_ and CH_4_ used during the process were at 1 and 0.1% concentrations, respectively, and this is significantly below the LEL (i.e., 4 and 5% for H_2_ and CH_4_, respectively). This allows this process to operate in safe conditions, and this can be easily implemented in an industrial setting (see the SI). Optimized process temperature and pressure were 980 °C and 780 mbar, respectively. Notably, no wire pretreatment was necessary, besides reactor annealing, prior to graphene growth. This is also relevant as most of the work reported to date requires some form of chemical pretreatment, thus introducing additional complexity and significant cost to the implementation of this graphene coating process at industrial premises. Table S1 presents a comparison between this work and those present in the literature with respect to sample chemical pretreatment, process temperature, and gas LEL.

**Figure 1:**
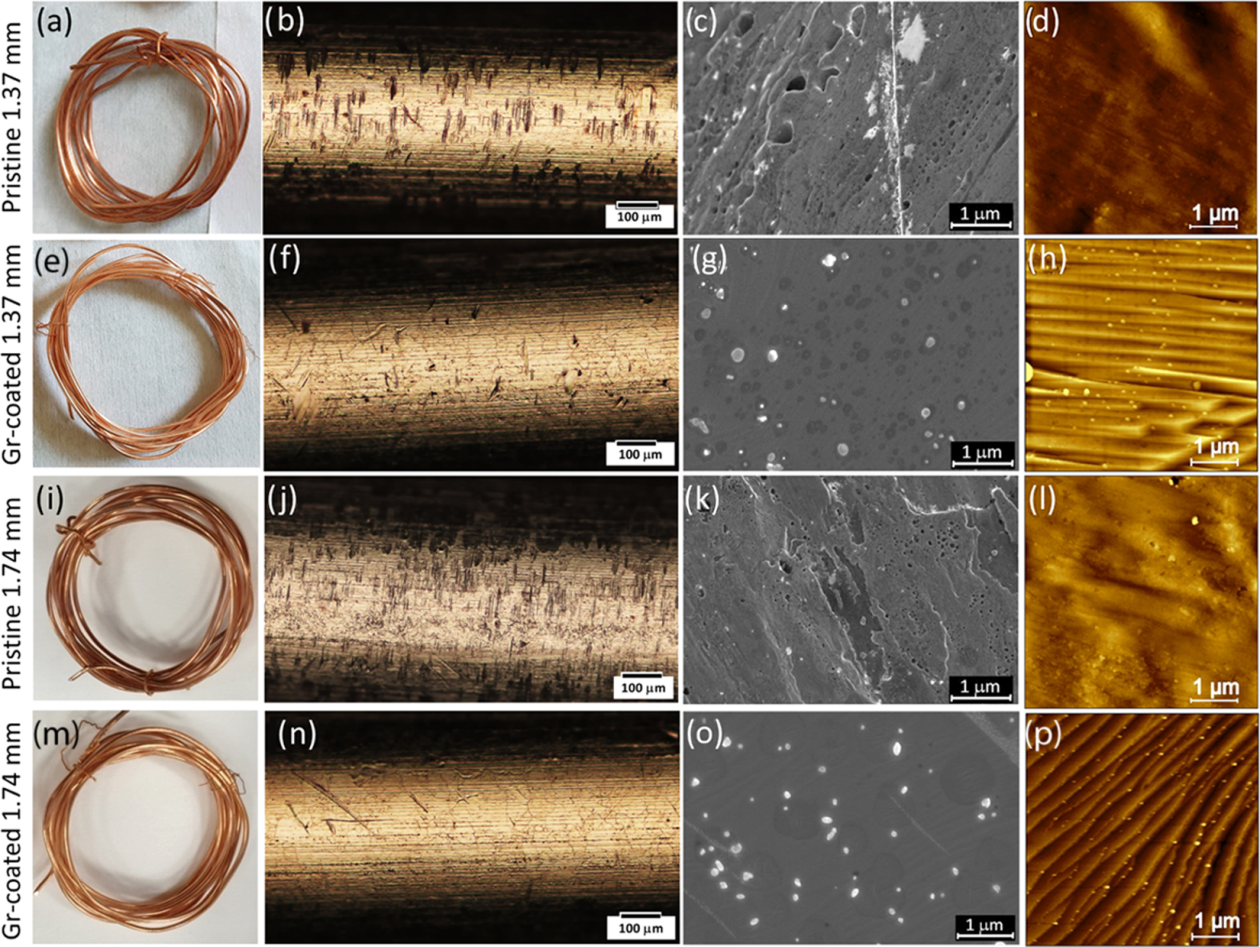
Morphological properties of pristine and Gr-coated Cu cables. Pictures of (a, i) pristine and (e, m) Gr-coated Cu wires arranged in coils. Optical micrographs of (b, j) pristine and (f, n) Gr-coated Cu wires. SEM micrographs of (c, k) pristine and (g, o) Gr-coated Cu wires. AFM topography images of (d, l) pristine and (h, p) Gr-coated Cu wires. All data are reported for both 1.37 and 1.74 mm wire diameters.

Figure 1 shows the appearance of the pristine (panels a, i) and Gr-coated (panels e, m) Cu wires, arranged in a coil shape. After the CVD process, the Gr-coated wires display higher malleability and appear shinier and smoother when compared to the pristine ones. The optical characterization of the wire surface, shown in Figure 1 (panels b, f, j, n), reveals no evident oxidation signs, both for the pristine and for the as-processed wires. It can be further observed that the Gr-coated wires display a smoother and more homogeneous surface compared to the pristine counterparts, for both diameters.

To study in detail the surface morphology, SEM and AFM characterizations for all the samples were performed. Figure 1c,d and k, l reports the SEM and AFM micrographs of pristine Cu wires, 1.74 and 1.37 mm diameters, respectively. For both diameters, an inhomogeneous surface can be observed, with RMS roughness values of 6.5 and 5.6 nm for 1.37 and 1.74 mm (over areas of 1 μm^2^), respectively. SEM micrographs of the Gr-coated wires display instead a smoother wire surface decorated with particles of ∼160 nm size. We verified that the presence of particles could be avoided by introducing a wire pretreatment step, i.e., Cu electropolishing^25^ (see Figure S6). AFM micrographs reveal the presence of atomic terraces, typically observed after the growth of graphene with good crystallinity and thickness homogeneity.^25−28^ The RMS roughness values measured from 1 μm^2^ micrographs for the 1.37 and 1.74 mm wires are ∼3.9 and 4.6 nm, respectively.

**Figure 2:**
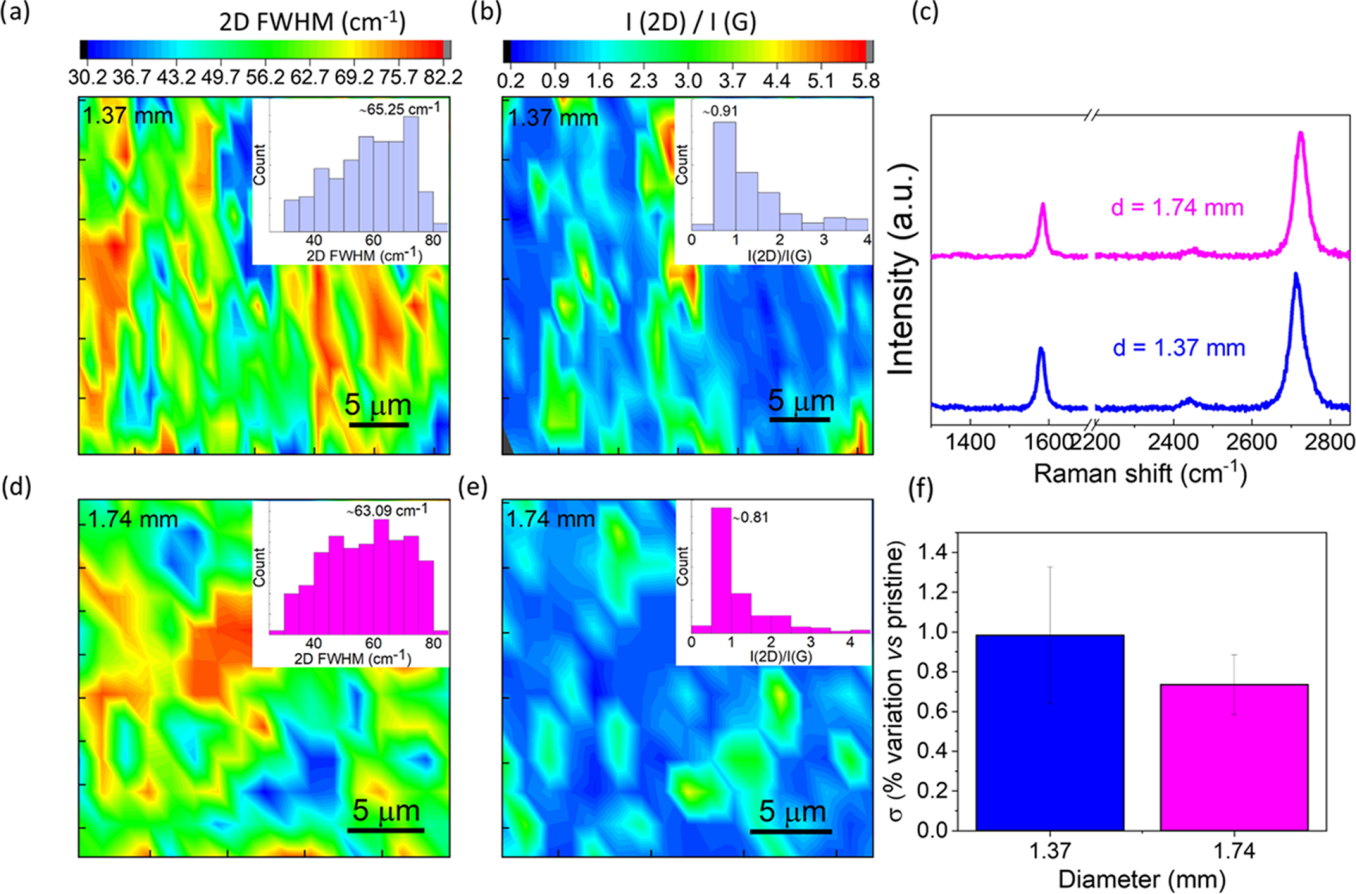
Spectroscopic and electrical properties of Gr-coated Cu wires. Representative Raman 30 × 30 μm^2^ maps (with relative histograms in the insets) reporting the (a, d) FWHM (2D) and (b, e) intensity ratio of 2D/G bands for both thicknesses. (c) Representative Raman spectra for 1.37 and 1.74 mm wires. (f) Electrical conductivity improvement of Gr-coated wires with respect to pristine ones. Error bars indicate the standard deviation of the mean (SDM).

Raman spectroscopy confirmed the presence of graphene films on the wires of both diameters. As seen in Figure 2c, sharp G (∼1590 cm^–1^) and 2D bands (2720 cm^–1^) could be visualized. The average *I*_2D_/*I*_G_ ratios over an area of 30 × 30 μm^2^ were found to be ∼0.9 and ∼0.80 for 1.37 and 1.74 mm diameters, respectively (Figure 2b,e). Similarly, the average FWHM (2D) values were measured as 65 and 63 cm^–1^ for 1.37 and 1.74 mm diameters, respectively (Figure 2a,d). Combined analysis of FWHM (2D) and *I*(2D)/*I*(G) indicated that the average number of layers is 2–3.^29,30^ Furthermore, the as-grown samples exhibited a negligible D peak (see Figure 2c), indicating a negligible number of defects. No sp2-related bands are observed for pristine and annealed wires in the same wavenumber range.

The electrical conductivity of Gr-coated wires measured at controlled temperature with a commercial system at industrial premises is reported in Figure 2f as a percentage variation with respect to pristine wires. Immediately after growth, Gr-coated wires displayed average improvements of 0.98% and 0.74% for 1.37 and 1.74 mm wires, respectively. Improvement of this magnitude could not be only related to the presence of the graphene conductive layer since in our geometry the conduction in graphene represents less than 10^–6^ the one in the copper, different from those in Kashani et al.^9^ and Kang et al.,^16^ where the copper cross section is three orders of magnitude smaller. It should be mentioned that already a 0.6% conductivity improvement would lead to a wire diameter reduction corresponding to a Cu cost reduction of 200€/Ton,^31,32^ an appealing saving for Cu cable manufacturers. In addition, we verified the mechanical resistance of graphene to manual handling by collecting Raman maps before and after rolling and unrolling operations of the coated wires. We did not observe any measurable variation in graphene quality and homogeneity, confirming the mechanical stability of the graphene coating (see Figure S7).

### 3.2 Aging of Gr-Coated Cu Wires

To assess the performances of aged Cu wires, a complete characterization of the samples by means of optical microscopy, Raman spectroscopy, and electrical measurements was carried out. The effect of aging on the chemical properties was investigated at different time points, from 6 to 24 months after the CVD process.

**Figure 3:**
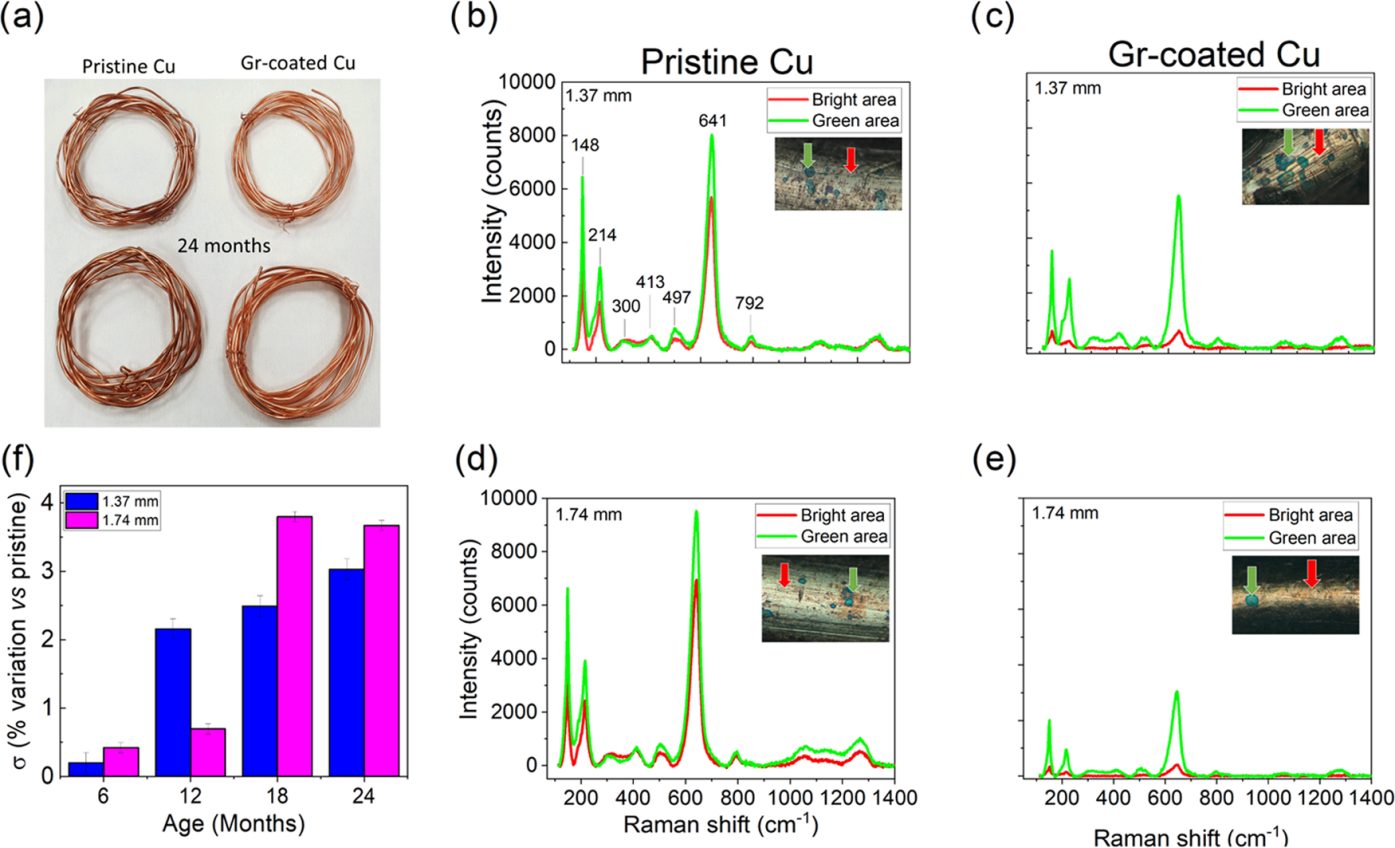
Characterization performed after aging of pristine and Gr-coated Cu wires.(a) Optical image of pristine and Gr-coated wires after 24 months. Raman spectra taken on different areas (indicated by arrows in the insets) of (b and d) pristine copper wires and (c and e) Gr-coated copper wires, for both thicknesses after 24 months. (f) Electrical conductivity improvement of 1.37 and 1.74 mm Gr-coated wires with respect to that of pristine wires from 6 to 24 months of aging.

Figure 3a shows a picture of pristine and Gr-coated Cu wires after 24 months of aging: color darkening—a clear sign of oxidation—is very evident in uncoated wires, while the Gr-coated ones maintain a shiny and new appearance. Figure S8 confirms a similar trend for both kinds of wires also at intermediate aging times. It is well known that Cu wires tend to become darker over time: this is usually thought as an indication of “poor quality” and causes significant sale returns, which negatively affects Cu wire manufacturers and retailers. Graphene coating offers a viable solution to this issue, providing an esthetic advantage.

Optical microscopy analysis might apparently contradict the resistance to oxidation of Gr-coated wires since oxidation spots (observed as green areas) can be found on both pristine and Gr-coated wires (see the insets in Figure 3b–e). However, Raman analysis confirms that Gr-coated wires have a higher oxidation resistance. Raman spectra were recorded in the copper oxide (Cu*_x_*O) range (between 150 and 800 cm^–1^),^14,32,36^ on both the green and bright areas (Figure 3b–d, c–e). Remarkable differences can be observed when comparing the uncoated and Gr-coated samples. The pristine wires (Figure 3b,d) display a very high intensity of Cu*_x_*O peaks both in the green and bright areas, demonstrating extended oxidation, which involves the entire surface of the wire. On the contrary, in the case of Gr-coated wires (Figure 3c,e) Cu*_x_*O peaks with a significantly lower intensity than those found in pristine wires are measured in the green areas, with the rest of the surface having a negligible Cu*_x_*O signal. Indeed, we notice that preferential paths for oxidation upon aging and/or exposure to extreme environmental conditions are defective regions such as those found in the correspondence of grain boundaries,^14,18^ Cu particles, or other types of defects that can open up percolative paths for contaminants (see Figures S9 and S10). It is to be noted that defective graphene (even 2/3 layer thick) is not effective in preserving Cu wires from oxidation and might even be counterproductive, owing to galvanic coupling, as previously reported in refs.^34,35^ The electrical properties of the wires at room temperature were also investigated for different aging times by recording their resistance at 10 mA. The relative improvement of the electrical conductivity, calculated with respect to the pristine Cu wires at the same aging stage, is reported in Figure 3f. The different electrical behavior for wires with and without graphene coatings over 6 to 24 months is apparent. After 12 months, a gradual worsening of the electrical properties for the pristine wires was observed, while the resistivity of the Gr-coated Cu wires remained comparable to that of the as-grown ones. This result further confirms that the presence of graphene significantly reduces the deterioration of Cu properties. Remarkably, after 24 months, relative improvements in conductivity of 3.0 and 3.6% are observed for the 1.37 and 1.74 mm Gr-coated samples, respectively. The waning of electrical conductivity in the pristine wires is associated to the oxidation of Cu discussed above.^18,33^ Furthermore, the graphene coating was found to be effective in preserving the wires from significant oxidation even at high temperatures (up to 90 °C) and humidity levels (up to 100%) (see Figure S9).

### 3.3 Effect of Wire Annealing and Hydrogen Treatment on Wire Microstructure

**Figure 4:**
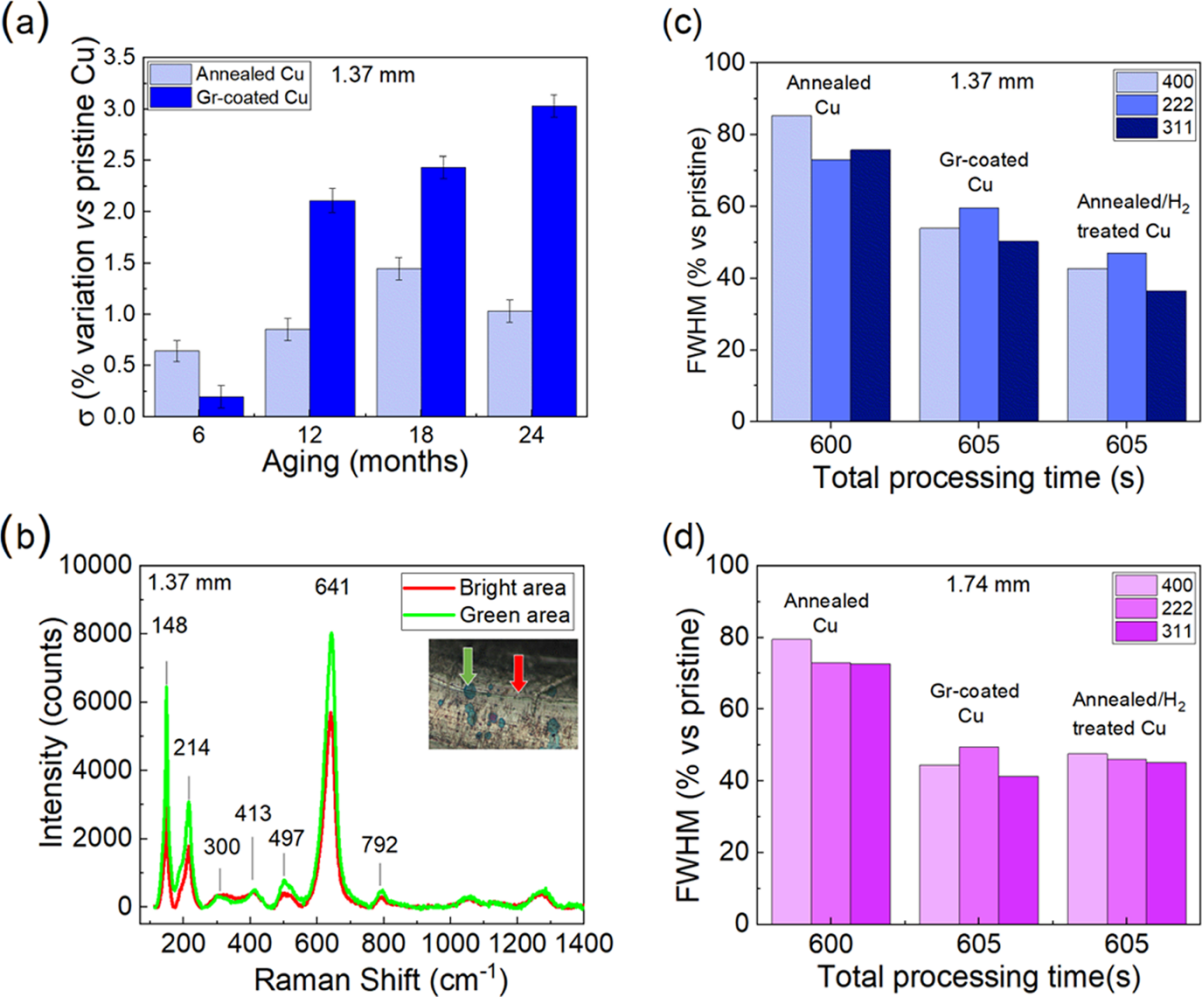
(a) Electrical conductivity improvement of annealed and Gr-coated Cu with respect to pristine wires from 6 to 24 months of age. (b) Raman spectra of annealed Cu taken in different areas of the wire (see the inset) after 24 months of aging in the Cu*_x_*O range. (c, d) FWHM evolution of (400), (222), and (311) diffraction peaks for Cu wires that were annealed, Gr-coated, and annealed plus H_2_-treated; both diameters were considered.

From the analyses reported above, it appears that Gr-coated wires have the following advantages with respect to pristine ones: (i) increased electrical conductivity at time zero and over time and (ii) oxidation protection. One can argue that the increase in electrical conductivity could be induced by microstructural changes in the Cu wires due to processing conditions rather than from the presence of the thin layer of graphene itself. Indeed, on millimeter-sized wires, it is quite unlikely that a thin layer of graphene could have such a remarkable effect on the electrical properties, although such an explanation has been provided for thinner wires.^9^ In order to assess the influence of thermal annealing on the electrical properties of Cu wires, the electrical conductivity of annealed wires over time was investigated. Despite exhibiting a shiny appearance (Figure S11) and improved conductivity at *t* = 0, it was found that the conductivity improvement over time is lower than that measured for Gr-coated wires (see Figure 4a). Hence, the presence of graphene seems pivotal for maintaining augmented electrical performances. Indeed, Raman analyses (as well as optical imaging) of aged wires indicate that oxidation progresses in annealed cables in a similar fashion to that in pristine ones (see Figures 3b and 4b). After 24 months, annealed wires appear significantly darker than the Gr-coated wires, and Cu*_x_*O peaks are comparable to those measured for pristine wires of the same age (Figure S12). With the aim of further investigating qualitative microstructural differences in pristine, annealed, and Gr-coated wires, XRD measurements were performed. The qualitative assessment of the XRD peaks further clarifies the role of annealing and graphene growth steps in the conductivity of copper. The histograms in Figure 4c,d report the relative variation (in %) of the FWHM of each peak with respect to the pristine samples, against the total duration of thermal treatment (the XRD patterns are shown in the SI, Figure S13). The annealed wires display narrower FWHM compared to the pristine Cu wires, and further FWHM reduction is observed upon Gr growth. The FWHM is sensitive to the variation in microstructure (crystallinity, presence of defects, and grain size) and stress–strain accumulation in the material.^37^ During the annealing and recovery process, the improved atomic diffusion at high temperatures enables the release of the stored strain energy of the extruded wires. The decrease of XRD peak broadening is suggestive of a decrease of crystallite strain, which can explain the enhanced conductivity of Cu after annealing. The growth step implies a remarkable decrease in peak width regardless of the annealing time (see the SI, Figure S14). This observation suggests that the conditions used for graphene coating relieve crystallite strain much more effectively than annealing in Ar does.

The aforementioned microstructural observations, as well as the increased malleability and smoothness, confirm that the enhanced conductivity is due to a more radical and deep change in the bulk copper crystallinity occurring during the growth treatment rather than to the presence of graphene itself. Since the growth step employs a mixture of H_2_/CH_4_, the effect of hydrogen on the microstructure of copper was investigated, analyzing samples annealed in Ar and then processed in H_2_ flow. The details of the samples and process conditions are reported in Table S2 of the SI. As demonstrated by the histograms reported in Figure 4 for a process where the standard annealing was associated with subsequent hydrogen treatment for 5 s (without concomitant graphene growth), the peak widths are comparable to those obtained after 5 s growth or possibly even narrower in the case of 1.37 mm wires.

Additional XRD measurements were performed for different annealing and hydrogen treatment times (see Figure S14 and Table S2), which further confirm that high-temperature H_2_ treatment positively affects Cu crystallinity.^38^ In conclusion, we can identify the hydrogen gas present in the CVD process during graphene growth as the main player in the improvement of Cu crystallinity and the consequent enhanced electrical properties; however, the structural, chemical, and electrical measurements performed for the different wires indicate that graphene is necessary for maintaining enhanced electrical properties over time.

### 3.4 Gr-Coated Cu Wires with an Ambient-Pressure R2R CVD Reactor

**Figure 5:**
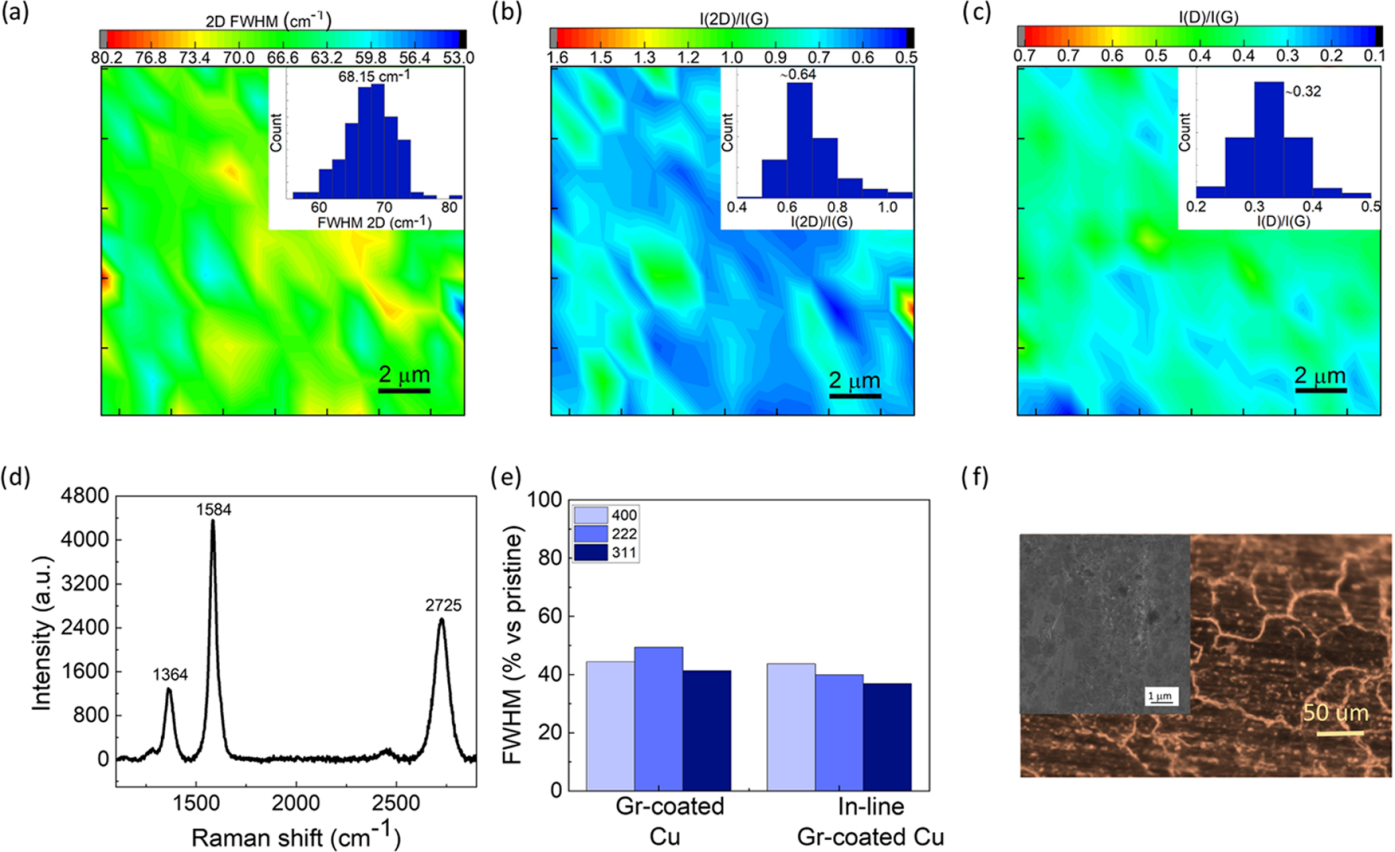
Characterization of Cu wires Gr-coated in an R2R prototype reactor. Representative Raman 25 × 25 μm^2^ maps (with relative histograms in the insets) reporting the (a) FWHM(2D) and intensity ratio of (b) 2D/G and (c) D/G bands. (d) Representative Raman spectrum of graphene grown on 1.74 mm Cu wire. (e) Comparison of FWHM evolution of (400), (222), and (311) diffraction peaks for Cu wires coated with graphene in the lab and the in-line reactor. (f) Optical image with the SEM micrograph in the inset.

The results above show that graphene coating of Cu wires is instrumental to obtaining combined improved electrical conductivity over time and oxidation resistance. This makes graphene an appealing coating for LV Cu wires, while graphene coating of metals might be of interest also for other applications spanning from nautical, electrical vehicles to aerospace.^39−42^ However, the scalable coating of metallic wires with graphene can be enabled only by the development of an ambient-pressure R2R CVD reactor. In this work, the properties of Gr-coated Cu wires obtained with such an R2R CVD reactor developed by AIXTRON Ltd. are presented. Using this in-line R2R CVD system, graphene was grown successfully onto a 1.74 mm Cu wire, and the relevant characterizations are reported in Figure 5. Raman spectroscopy indicates a uniform few-layer graphene film. The FWHM (2D) averages at ∼68 cm^–1^, the 2D/G intensity ratio is found to be ∼0.64. and the D/G intensity ratio is ∼0.33 (see Figure 5a–c). XRD measurements reveal an increased Cu crystallinity, comparable to that of Gr-coated wires processed in a batch reactor (see Figures 4d and 5e). Optical and SEM characterizations confirm the presence of a uniform coating across the surface (Figure 5f). These results indicate that the process developed in laboratory settings can be implemented in an industrial in-line system with promising results.

## 4 Conclusions

In this work, we report an industrially compatible CVD process to coat with graphene electrical Cu wires with diameters relevant to LV applications. The Gr-coated wires display improved aesthetics and enhanced electrical conductivity with respect to uncoated (pristine) wires. Specifically, the conductivity is 1% higher than that of pristine wires immediately after graphene coating and 3% higher after 24 months. Structural analyses performed via XRD indicate that the exposure to high temperatures and the presence of hydrogen during graphene growth cause an increase in Cu crystallinity, which is a reasonable explanation for the observed increase in electrical conductivity. Combined electrical and chemical characterizations show that graphene efficiently acts as a barrier against oxidation (even under different environmental conditions), which is beneficial to preserve the augmented conductivity in time. The reported increase in electrical conductivity potentially allows for a reduction in wire diameter with consequent savings in wire production costs. Building on such promising premises, we demonstrate that the developed process can be adopted in a R2R CVD reactor for in-line coating of metallic wires, which builds a road towards the realistic translation of the graphene coating technology in industrial settings. The extension of such coating technology also to flexible Cu cables and wires adopted in automotive and aerospace industries could lead to a significant decrease in the overall amount of Cu weight, leading to lighter vehicles with a lower impact on the environment.

## Supporting Information Available

Experimental and measurement setups, including photographs of the experimental setup and Cu wires, tables, optical images, XRD patterns, SEM images, and Raman analysis results (PDF)
