# Supplementary material for: Industrial Graphene Coating of Low-Voltage Copper Wires for Power Distribution
Source: ACS Appl Eng Mater. 2023 Jun 1;1(7):1937–45. doi: 10.1021/acsaenm.3c00249 (PMC10391742; doi:10.1021/acsaenm.3c00249)
Supplement: Supplementary file 1 — em3c00249_si_001.pdf [file em3c00249_si_001.pdf]

Supporting Information

Industrial Graphene Coating of Low-Voltage Copper Wires for Power Distribution

Neeraj Mishra*#[1,2], Ylea Vlamidis*[1,2], Leonardo Martini[1,2], Arianna Lanza[1], Zewdu M. Gebeyehu[1,2], Alex Jouvray[3], Marco La Sala[4], Mauro Gemmi[1], Vaidotas Mišeikis[1,2], Matthew Perry[3], Kenneth B.K. Teo[3], Stiven Forti[1], Camilla Coletti[1,2,#]

*Equal contribution

#corresponding Author: neeraj.mishra@iit.it ; camilla.coletti@iit.it

[1]Center for Nanotechnology Innovation@NEST, Istituto Italiano di Tecnologia, Piazza San Silvestro, 12−56126 Pisa, Italy

[2]Graphene Labs, Istituto Italiano di Tecnologia, Via Morego 30, 16163 Genova, Italy

[3]AIXTRON LTD, Anderson Road, Swavesey, Cambridge CB24 4FQ, United Kingdom

[4]Baldassari Cavi, Viale Europa 118/120, 55013 Capannori (Lucca), Italy

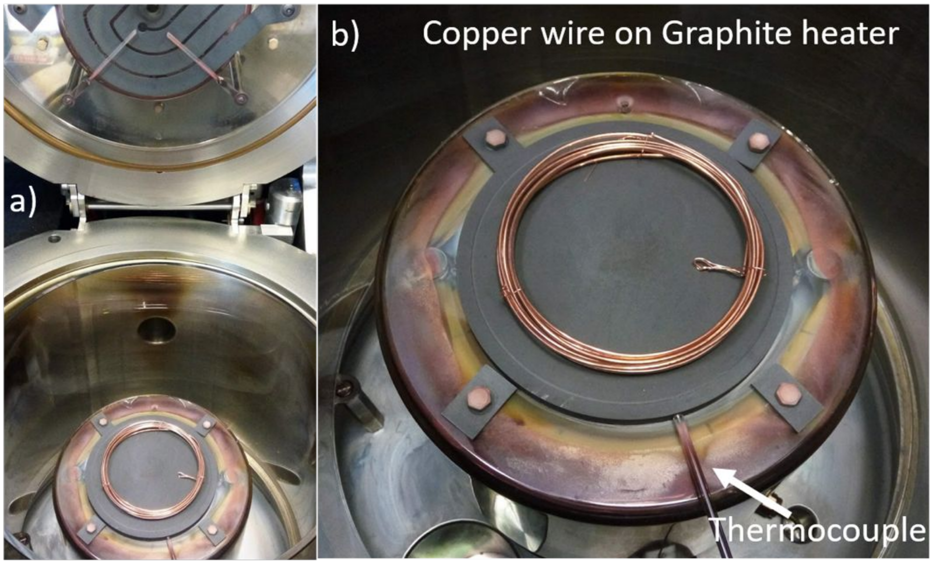

Figure S1. Copper (Cu) coil placement within the 4-inch cold wall AIXTRON BM reactor. Inside view of the reactor with lid (a), featuring a top heater and inlet for the gases, visible. Zoom-in image showing the Cu coil on top of the graphite heater (b). The thermocouple is indicated by an arrow.

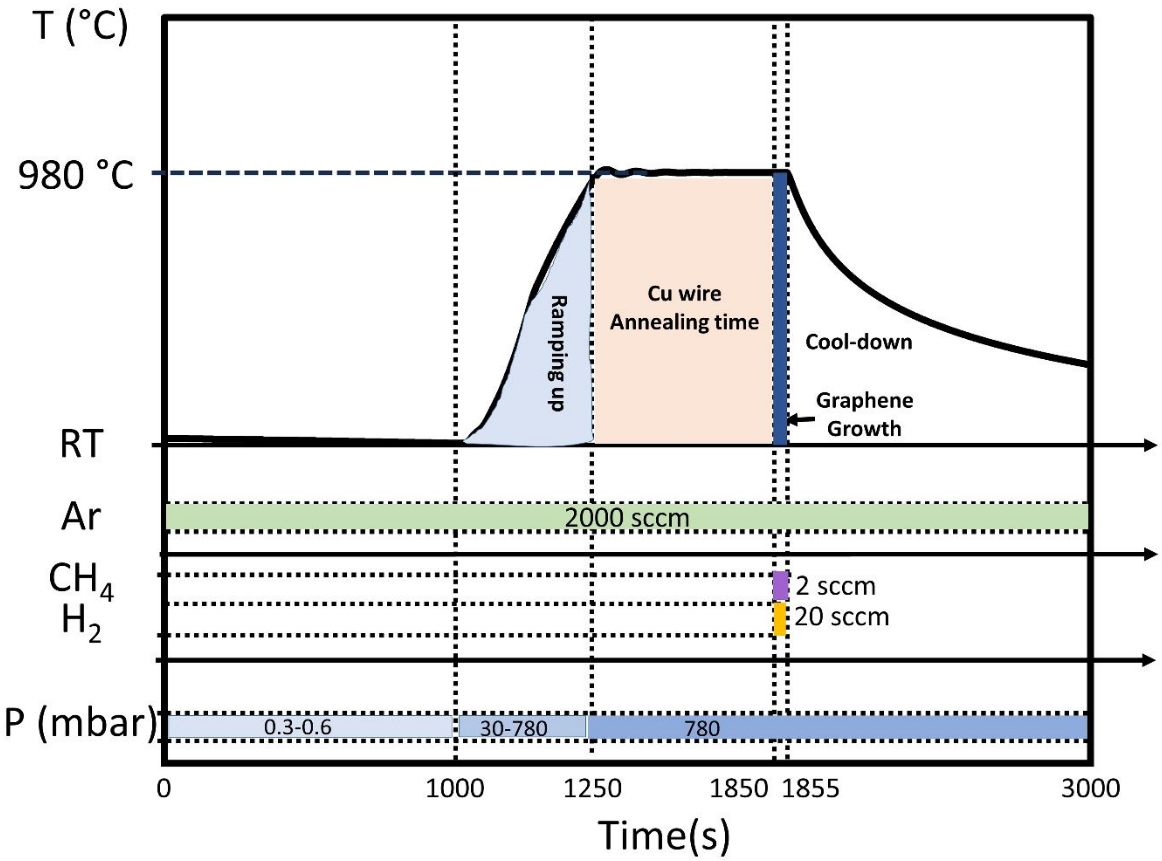

Figure S2. Schematic diagram showing the temperature profile and conditions employed in the CVD process.

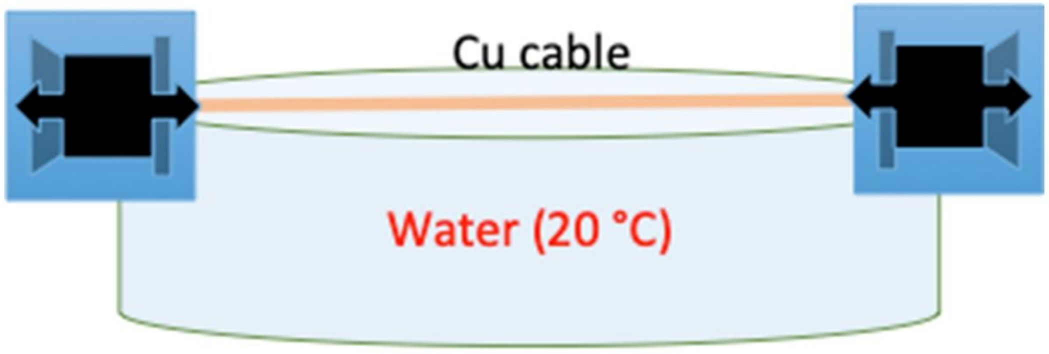

Figure S3. Standard set-up for resistivity measurements at 20 °C.

CVD growth process optimization

Graphene was grown on copper wire of both diameters (1.37 mm and 1.78 mm) at different temperatures, pressures, gas fluxes, annealing and growth times. Concerning pressure, 780 mbar were found to be an ideal compromise to obtain graphene growth at nearly atmospheric pressure. Argon gas was used during the annealing step to increase Cu grain size.[1] Annealing times adopted during the preliminary experiments were 0, 5, 10 and 30 min (Figure S4) at different temperatures (900, 930, 950, and 980 °C). The annealing step was found to improve surface morphology and promote Cu terrace formation. An annealing time of 10 min at 980 °C was found to be a good compromise, yielding well-defined terraces when compared to 0 s and 5 min annealing, while avoiding lengthy processing times (Figure S4). Adopted growth temperatures were 900, 930, 950, and 980 °C (Figure S5). Growth temperatures lower than 950 °C yielded to the formation of amorphous carbon for all the growth times adopted (0 s, 1 s, 5 s, 10 s, 5 min, 10 min, 30 min) as found by Raman spectroscopy analyses (not reported). A growth temperature of 980 °C for 5 s was found to yield continuous graphene. The SEM micrographs reported in Figure S5 show a significant improvement of the Cu surface morphology, instrumental for the growth of good quality graphene, with increasing growth temperature.

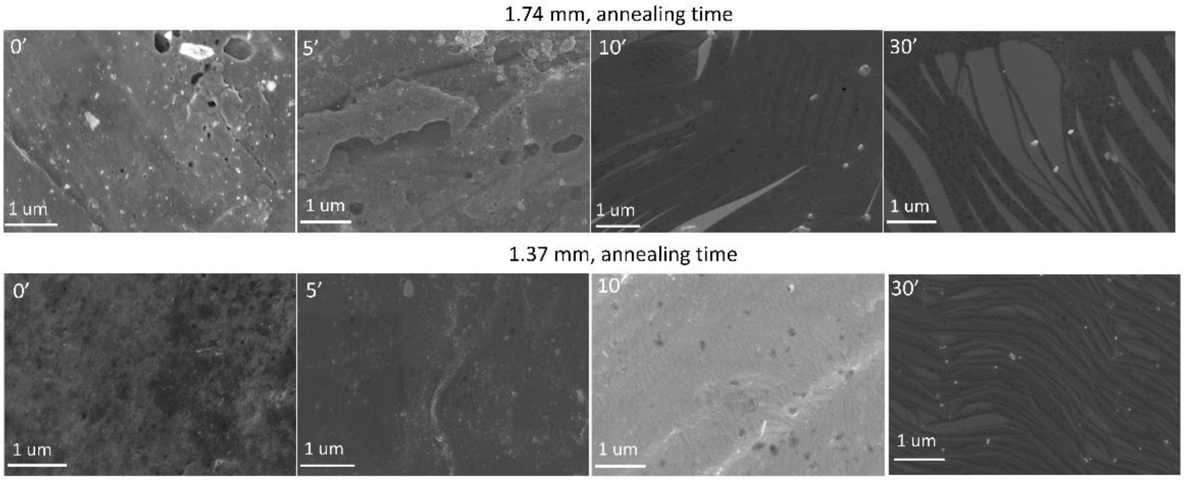

Figure S4. SEM images of copper wire of both diameters (i.e., 1.74 mm and 1.37 mm) annealed at 980°C in Ar atmosphere for 0 min, 5 min, 10 min and 30 min.

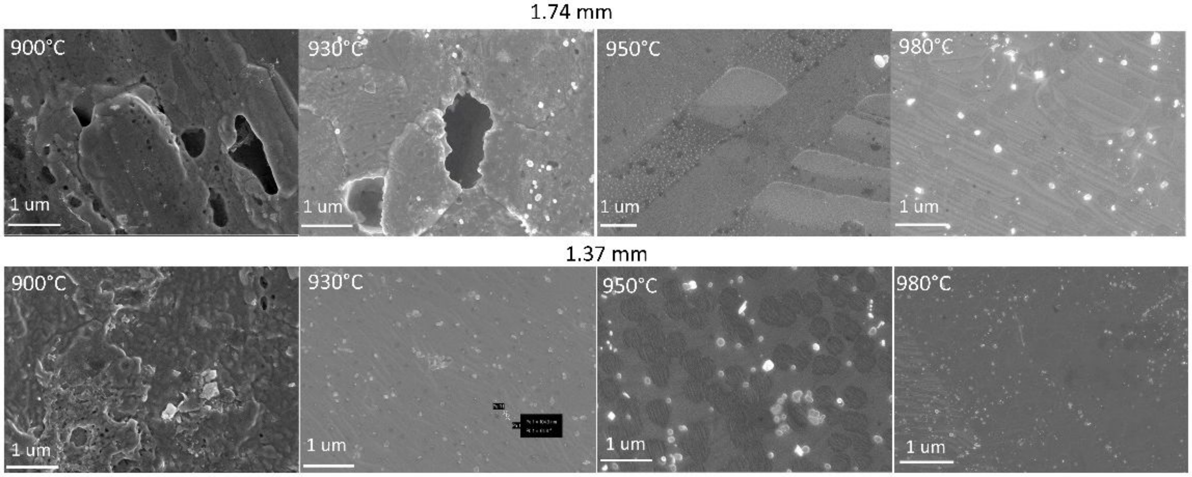

Figure S5. SEM images of copper wires of both diameters (i.e., 1.74 mm and 1.37 mm) after graphene growth for 5 s at temperatures of 900, 930, 950 and 980°C.

Lower and upper explosive limits

From an industrial point of view, to comply with safety requirements, it is important to maintain the formation of explosive gas mixtures below well-defined limits. Keeping this in mind, we optimized the flow of H_2 and CH_4 to be within the lower explosive limit (LEL) that is the lowest concentration of an explosive gas which will burn or explode if ignited. The LEL is determined empirically for each pure chemical and air mixture at a given temperature. If more than one chemical is dispersed in the air, as is normally the case, then Le Chatelier's mixing rule can be applied to get the cumulative LEL for the mixture.[2,3] Concentrations lower than the lower explosive limit are 'too lean' to burn; those above the Upper Explosive Limit (UEL) are too rich to burn. The amount of gas present is specified as a percentage (%) of LEL. Zero percent Lower Explosive Limit (0% LEL) denotes a combustible gas-free atmosphere.[2,3] The LEL values for H_2 and CH_4 are 4% and 5%, respectively.[3,4] Since the % volume used in our reactor is 1% and 0.1% for H_2 and CH_4, respectively, we operate in safe conditions, employing concentrations lower than the explosive limit.

Table S1. Comparison of process parameters employed in related works.

Reference	Pre-treatment	Process T (°C)	Gas flow (sccm)			Explosive gases (% vol.)	
			Ar	H_2	C source	H_2	C source
Lee et al.[5]	Ammonium persulfate	1050		10	0.1 (CH_4)	-	-
Jang et al.[6]	Ammonium persulfate	1000	500	100	2 (CH_4 or acetylene)	16.6 (> LEL)	0.3 (< LEL)
Datta et al.[7]	Electropolishing	1000	100	500	6 (CH_4)	82.50 (> UEL)	1 (<LEL)
Kashani et al.[8]	/	975-1000	1500	100	2-20 (Benzene)	6.3 (> LEL)	0.1-1.2 (<LEL)
This work	/	980	2000	20	2 (CH_4)	1 (<LEL)	0.1 (<LEL)

Electropolishing of Cu wires

Cu wires adopted in industrial plants present particles that could hinder the formation of a continuous graphene film. Pin hole gaps generated in the graphene film by the presence of particles on the copper surface will reasonably represent a preferential path for moisture to penetrate below the graphene coating and thus oxidize the copper wire. This problem can be addressed by electropolishing the copper wire with a procedure that is typically adopted for copper foils[1]. Figure S6 reports SEM and optical micrographs that show the effectiveness of the electropolishing process.

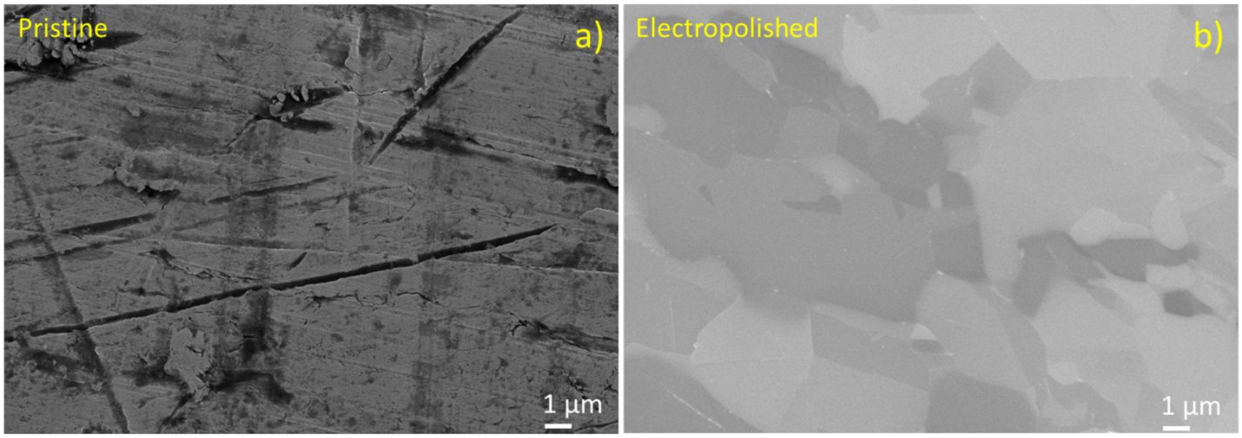

Figure S6. Morphological characterization of pristine Cu wires before and after electropolishing. Figure (a and b) shows SEM images of pristine Cu wire before, and after electropolishing. The procedure adopted is reported in[1], and specifically it was applied a voltage of 12V for 30s.

Graphene stability upon manual handling and mechanical operations

Mechanical stability of the graphene coating upon manual handling of the wires was verified by Raman spectroscopy performed in various sections of the wires (see Figure S7). Each 2-meter-long graphene coated Cu wire was inspected before and after rolling/unrolling operation. Figure S7 reports representative Raman maps taken for a freshly coated graphene wire and the same wire after 4 rolling/unrolling operations. All the maps recorded in different parts of the cable show similar features. No measurable variation was observed in the quality and homogeneity of graphene, suggesting a good stability of the coating upon mechanical solicitation. Furthermore, it should be mentioned that copper wires are typically coated with a polymeric (PVC) layer before being adopted in housing and industrial installations. Such layer indubitably provides an additional protection to the graphene coating.

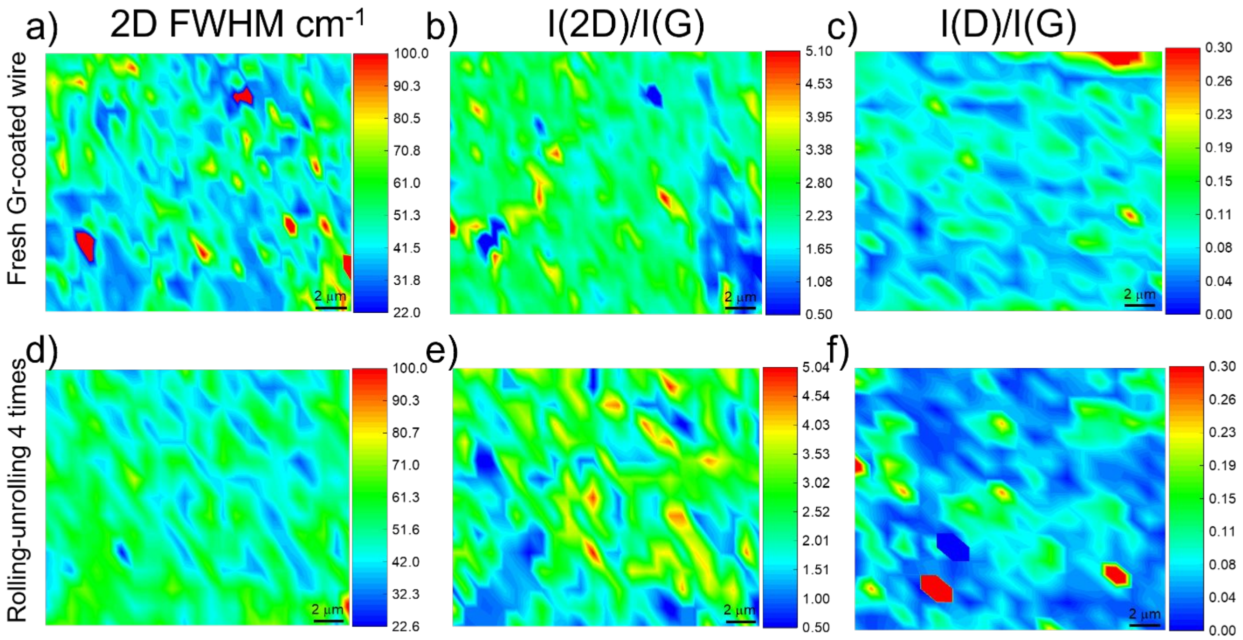

Figure S7. Raman characterization of the same freshly grown Gr-coated Cu wire measured before and after 4 rolling/unrolling operations. Panels (a and d), (b and e) and (c and f) show 2D FWHM, I(2D)/I(G), and I(D)/I(G), respectively.

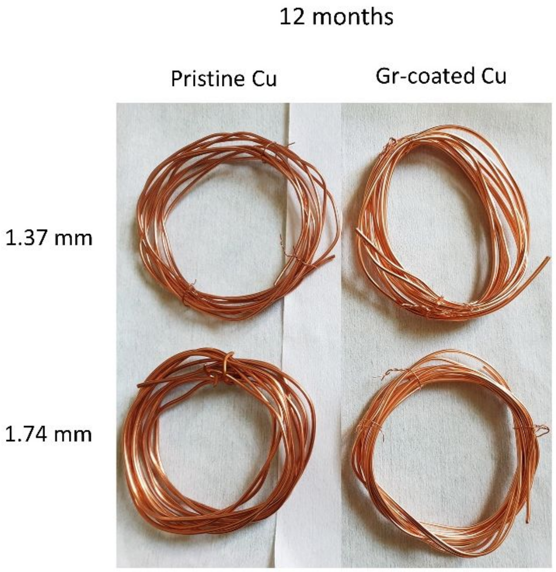

Figure S8. Optical images of pristine and Gr-coated Cu wires at 12 months.

Oxidation resistance experiments in the climate chamber

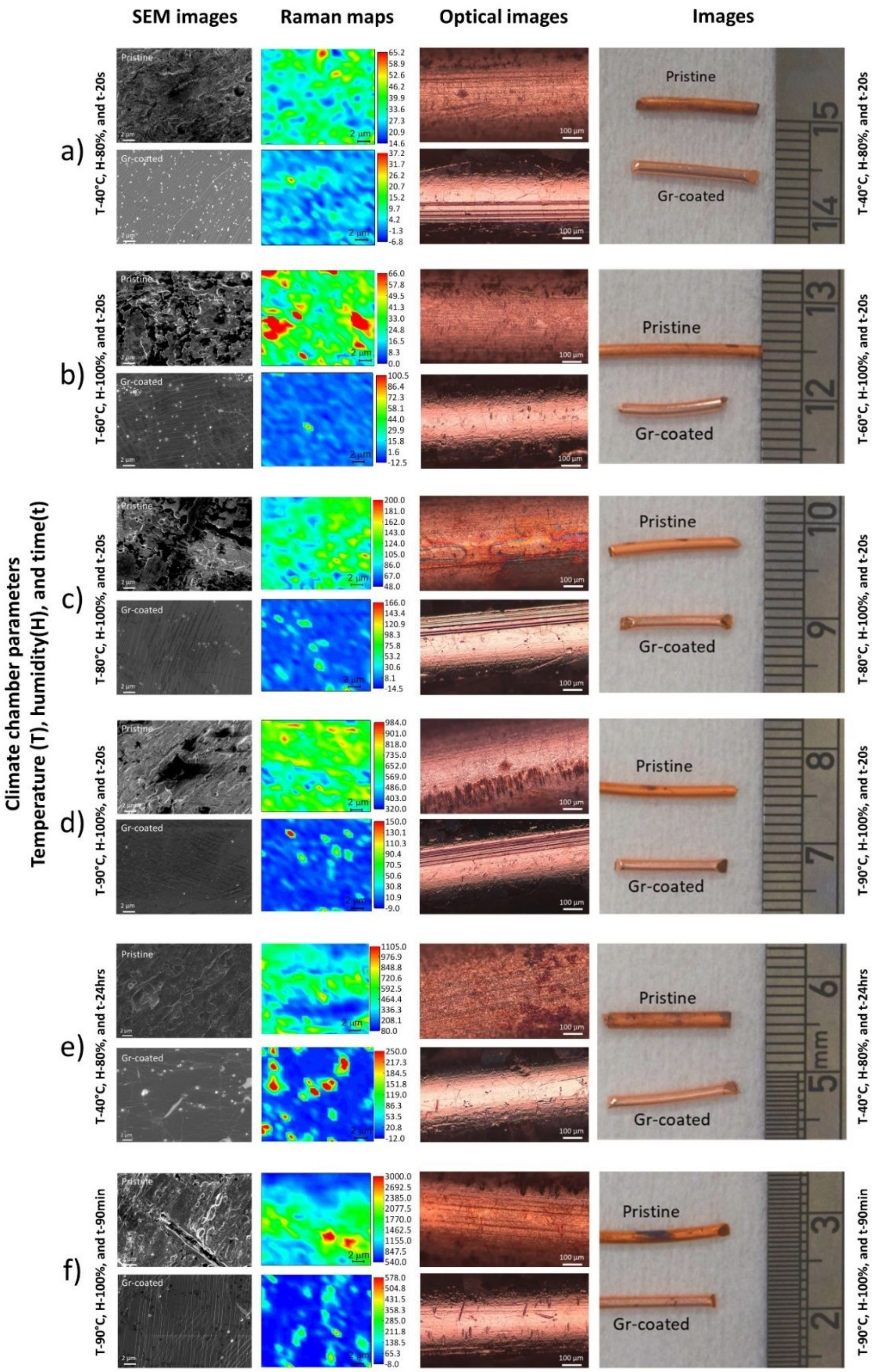

Figure S9. Characterization and comparison of pristine and Gr-coated Cu wires after they were kept in a climate chamber at different temperature (T), humidity % (H) and time (t) for testing their oxidation resistance.

The following experiments were performed to test the oxidation resistance of pristine and Gr-coated wires:

1. 40°C, 80%, and 20s
2. 60°C, 100%, and 20s
3. 80°C, 100%, and 20s
4. 90°C, 100%, and 20s
5. 40°C, 80%, and 24 hrs
6. 80°C, 100%, and 90 min

The climate chamber parameters were selected to simulate the extreme conditions that the wires could be subjected to during industrial processing. The treated Cu wires were characterized by SEM, Raman spectroscopy, and optical microscopy (see Figure S9). Already at an optical inspection the wires coated with graphene displayed a much shinier (less oxidized) appearance, even in the most extreme conditions. This qualitative optical assessment was quantitatively confirmed by Raman spectroscopy. In particular, when analyzing Raman spectra in the Cu_xO range (between 150 and 800 cm^{-1}) and when specifically plotting the intensity of the Cu_xO peak at 637 cm^{-1}, it becomes apparent that graphene is effective in protecting the wire from oxidation in every analyzed environmental condition. Even for the most extreme conditions we observe that graphene is effective in protecting the wire from massive and homogenous oxidation, as only localized spots appear to be oxidized (most of the map maintains a blue color).

To investigate the difference in oxidation protection behavior between the graphene coatings reported in the manuscript and defective graphene coatings of the same thickness (i.e., 2/3 layers), we purposedly coated Cu wires with defective graphene (average D/G ratio of 0.95) and subjected them to the same environmental conditions reported in Figure S9. As shown in Figure S10, Raman analyses (as well as optical microscopy and SEM, data not shown) demonstrate that such defective graphene coatings are not effective in protecting graphene from oxidation and in some cases galvanic coupling was observed. Instead, the graphene coatings discussed in the manuscript were confirmed to be effective as also demonstrated in Figure S9.

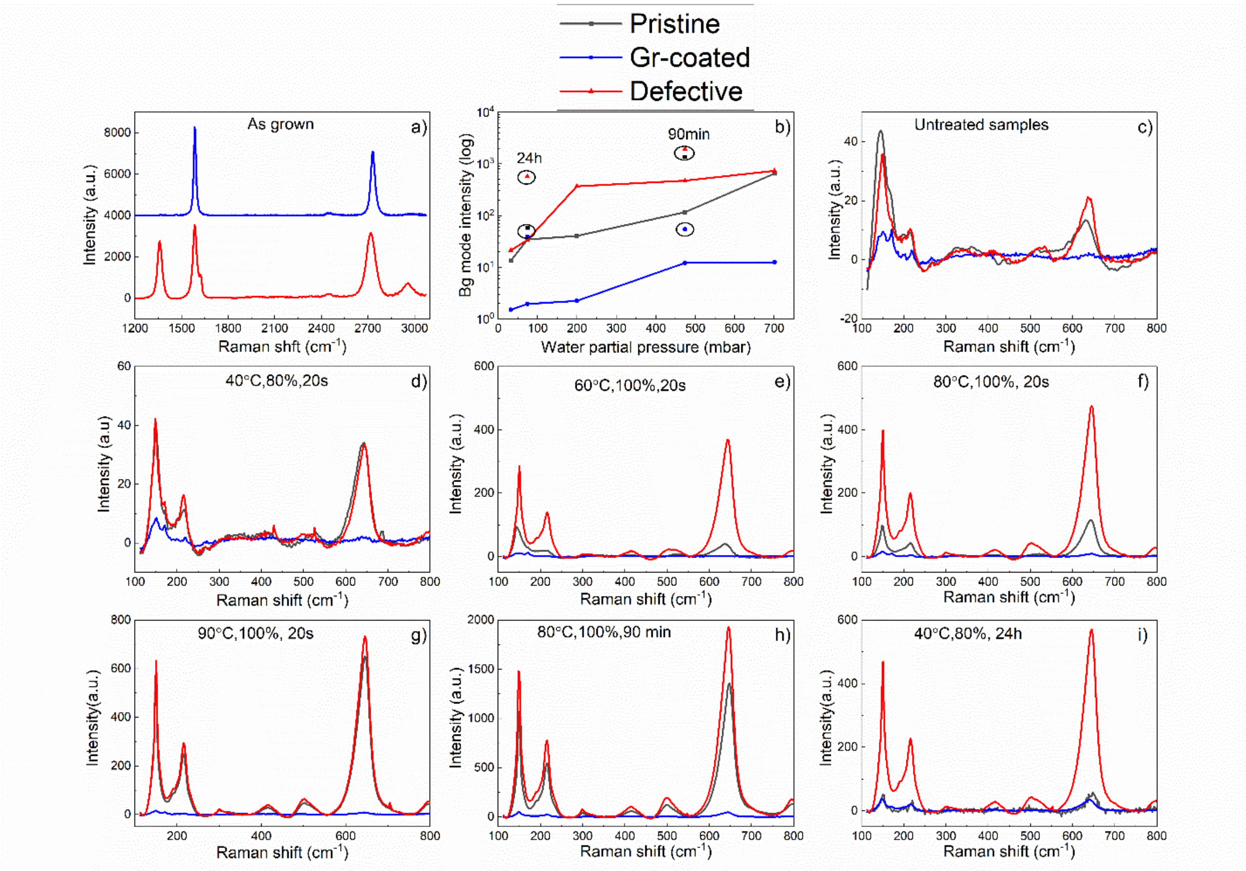

Figure S10. Comparative study of pristine (black line), few-layers Gr-coated (blue line), and defective few layers Gr-coated Cu wires (red line), respectively, subjected to different temperature (T), humidity % (H) and time (t) for testing their oxidation resistance in the climate chamber. a) Raman spectra of as grown few layers non-defective and defective graphene adopted in this experiment; b) Cu_xO Bg mode peak (637 cm^{-1}) plotted against water partial pressure (the data points inside the circle were obtained for 90 minutes and 24 hours, while those connected by a line where obtained for 20 seconds exposure; and (c-i) comparative Raman spectra measured in the Cu_xO range (150-800 cm^{-1}) after the climate chamber experiments.

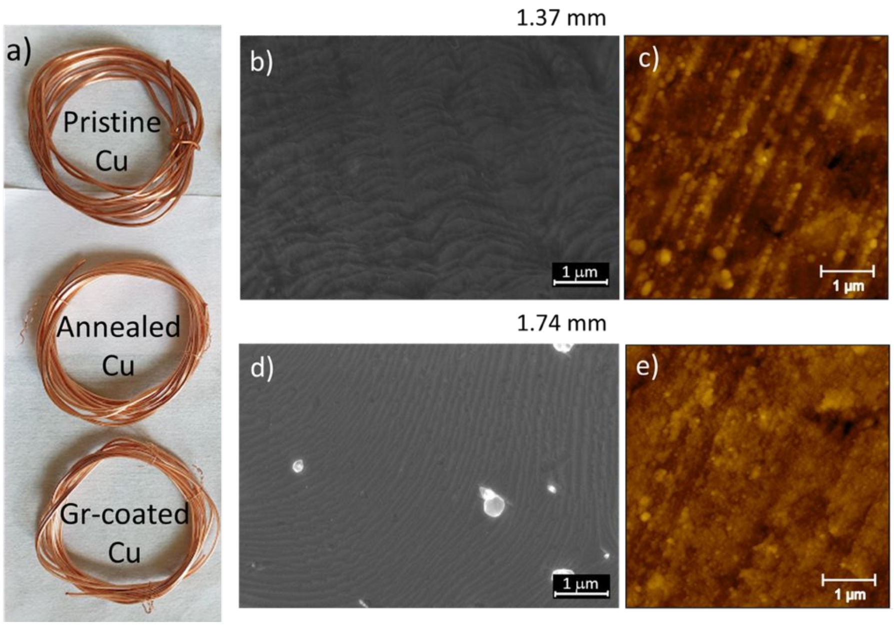

Figure S11. Optical (a), SEM (b, d) and AFM images (c, e) of annealed Cu wires. Both diameters are reported.

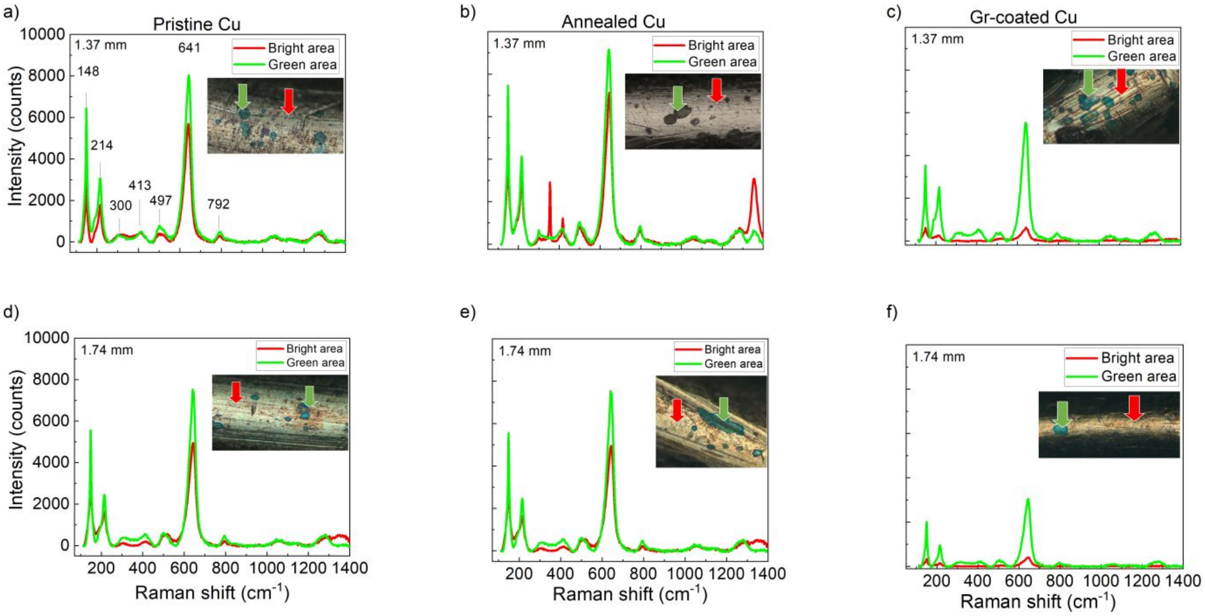

Figure S12. Comparison between Raman spectra recorded in the green areas and in the brighter areas for the pristine Cu (a, d), the annealed Cu (b, e) and grown samples (c, f) of both diameters after 24 months.

Table S2. Samples analyzed by XRD and related parameters and process conditions.

Sample	Annealing		Growth/H_2 atmosphere				Total time (s)
	Ar flow (sccm)	Time (min)	Gas flow (sccm)			Time (s)	
			CH_4	H_2	Ar		
pristine	---	---	---	---	---	---	0
0'/1" H_2	---	---	---	20	2000	1	1
0'/5"	---	---	2	20	2000	5	5
0'/5" H_2	---	---	---	20	2000	5	5
1'/5"	2000	1	2	20	2000	5	65
5'/0"	2000	5	---	---	---	---	300
5'/5"	2000	5	2	20	2000	5	305
10'/0"	2000	10	---	---	---	---	600
10'/1"	2000	10	2	20	2000	1	601
10'/5"	2000	10	2	20	2000	5	605
10'/5" H_2	2000	10	---	20	2000	5	605
10'/30"	2000	10	2	20	2000	30	630
30'/0"	2000	30	---	---	---	---	1800

XRD experiments

As shown in Figure S13, the FWHM decreases sharply for just 1s growth, and becomes extremely narrow for 5s growth, while by further prolonging the growth duration (30s) no appreciable differences are observed compared to 5s. Considering samples annealed for 10 minutes and grown for 5s the observed FWHM are roughly half with respect to those of the pristine samples. All peak families of for both wire thicknesses are aligned with this trend.

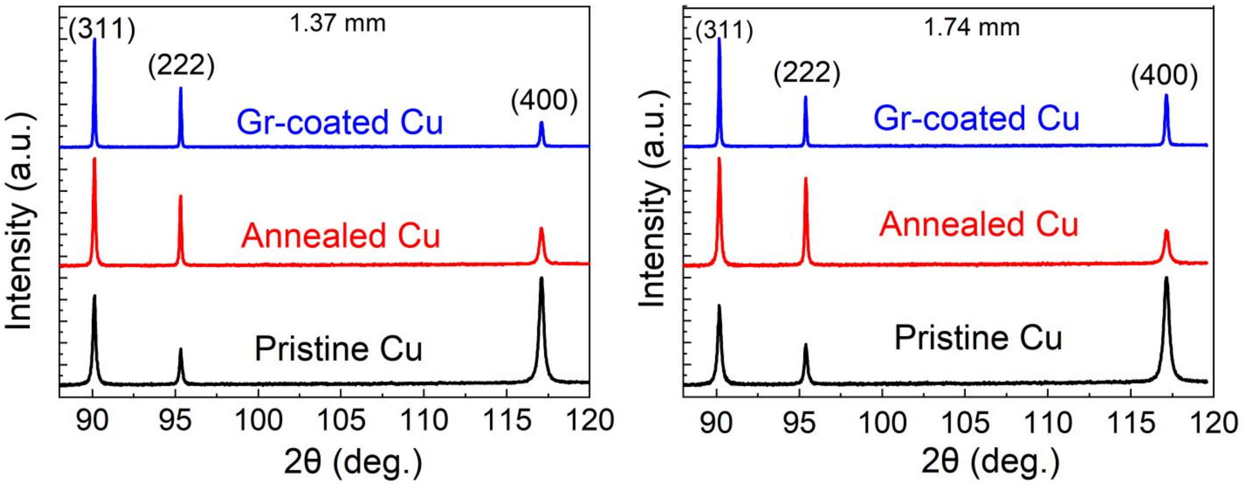

Figure S13. Representative XRD patterns of pristine and processed Cu wires, normalized to the highest intensity value (a: 1.37 mm and b: 1.74 mm size).

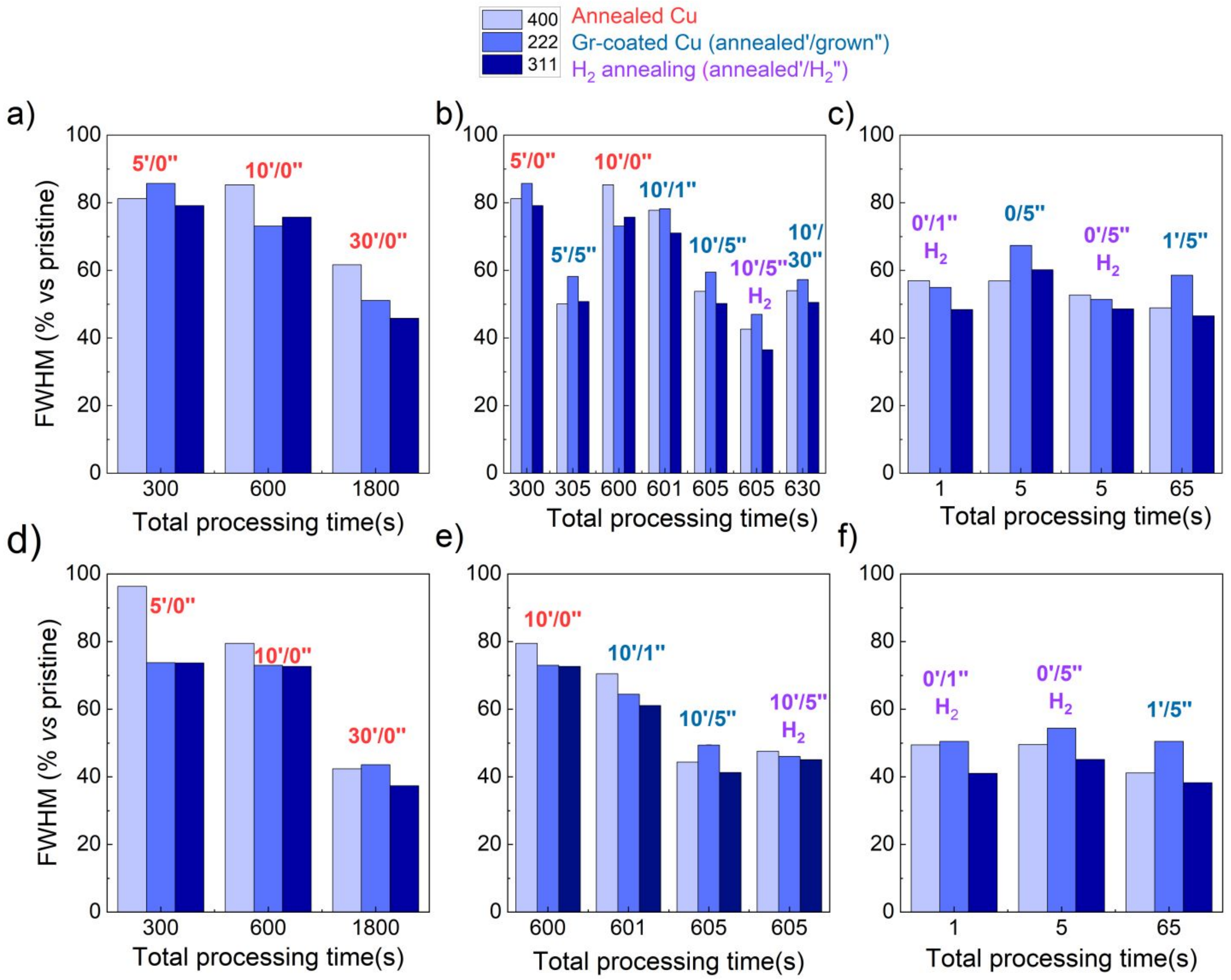

Figure S14. FWHM evolution of (400), (222) and (311) diffraction peaks for Cu submitted to different processing times: annealed samples (a, d), comparison between Gr-coated samples (annealed/grown) and wires annealed/processed in H_2 atmosphere (b, e), comparison between samples processed in hydrogen atmosphere and grown samples (c, f). Notation: annealed'/growth'' or annealed'/H_2". Both diameters were considered (a-c: 1.37 mm, d-f: 1.74 mm).

References

(1) Miseikis, V.; Convertino, D.; Mishra, N.; Gemmi, M.; Mashoff, T.; Heun, S.; Haghighian, N.; Bisio, F.; Canepa, M.; Piazza, V.; Coletti, C. Rapid CVD Growth of Millimetre-Sized Single Crystal Graphene Using a Cold-Wall Reactor. *2D Mater.* **2015**, *2* (1). https://doi.org/10.1088/2053-1583/2/1/014006.

(2) De Heer, J. The Principle of Le Chatelier and Braun. *J. Chem. Educ.* **1957**, *34* (8), 375–380. https://doi.org/10.1021/ed034p375.

(3) Wu, X.; Zhong, G.; D'Arsié, L.; Sugime, H.; Esconjauregui, S.; Robertson, A. W.; Robertson, J. Growth of Continuous Monolayer Graphene with Millimeter-Sized Domains Using Industrially Safe Conditions. *Sci. Rep.* **2016**, *6* (February), 2–8. https://doi.org/10.1038/srep21152.

(4) Zhong, G.; Wu, X.; D'Arsie, L.; Teo, K. B. K.; Rupesinghe, N. L.; Jouvray, A.; Robertson, J. Growth of Continuous Graphene by Open Roll-to-Roll Chemical Vapor Deposition. *Appl. Phys. Lett.* **2016**, *109* (19). https://doi.org/10.1063/1.4967010.

(5) Lee, B.; Li, W. Performance of Different Layers of Graphene as Protective Coating for Copper Wire. *Mater. Lett.* **2020**, *273*, 127875. https://doi.org/10.1016/j.matlet.2020.127875.

(6) Jang, L. W.; Zhang, L.; Menghini, M.; Cho, H.; Hwang, J. Y.; Son, D. I.; Locquet, J. P.; Seo, J. W. Multilayered Graphene Grafted Copper Wires. *Carbon N. Y.* **2018**, *139*, 666–671. https://doi.org/10.1016/j.carbon.2018.07.033.

(7) Datta, A. J.; Gupta, B.; Shafiei, M.; Taylor, R.; Motta, N. Growth of Graphene on Cylindrical Copper Conductors as an Anticorrosion Coating: A Microscopic Study. *Nanotechnology* **2016**, *27* (28). https://doi.org/10.1088/0957-4484/27/28/285704.

(8) Kashani, H.; Kim, C.; Rudolf, C.; Perkins, F. K.; Cleveland, E. R.; Kang, W. An Axially Continuous Graphene–Copper Wire for High-Power Transmission: Thermoelectrical Characterization and Mechanisms. *Adv. Mater.* **2021**, *33* (51), 1–14. https://doi.org/10.1002/adma.202104208.
